# Reference gene screening of *Batrachochytrium dendrobatidis* and *Batrachochytrium salamandrivorans* for quantitative real-time PCR studies

**DOI:** 10.1038/s41598-019-54582-4

**Published:** 2019-12-06

**Authors:** Elin Verbrugghe, Frank Pasmans, An Martel

**Affiliations:** Faculty of Veterinary Medicine, Department of Pathology, Bacteriology and Avian Diseases, Ghent University, Salisburylaan 133, 9820 Merelbeke, Belgium

**Keywords:** Fungal pathogenesis, Pathogens

## Abstract

Real-time quantitative PCR studies largely depend on reference genes for the normalization of gene expression. Stable reference genes should be accurately selected in order to obtain reliable results. We here present a study screening commonly used reference genes (*TEF1F*, *α-centractin*, *Ctsyn1*, *GAPDH*, *R6046*, *APRT* and *TUB*) in the chytrid fungi *Batrachochytrium dendrobatidis* (*Bd*) and *Batrachochytrium salamandrivorans* (*Bsal*), which cause the lethal amphibian skin disease chytridiomycosis. We evaluated the stability of the reference gene candidates during different growth stages of the fungi, using different statistical software packages: ΔCT, BestKeeper, GeNorm, NormFinder and RefFinder. In order to reflect the *in vivo* situation, the stability of the candidates was assessed when taking all growth stages into account. Using an *ex-vivo* approach, we tested whether the expression of *GAPDH*, *TUB*, *R6046* and *APRT* (*Bd*) and *GAPDH*, *TUB*, *R6046* and *α-centractin* (*Bsal*) remained stable when these fungi came in contact with host tissue. Finally, their role as *in vivo* reference genes was examined in skin tissue of experimentally infected midwife toads (*Alytes obstetricans*) (*Bd*) and fire salamanders (*Salamandra salamandra*) (*Bsal*). Summarized, the present study provides guidance for selecting appropriate reference genes when analyzing expression patterns of these fungal organisms during different growth stages and in *Bd*- or *Bsal*-infected tissues.

## Introduction

*Batrachochytrium dendrobatidis* (*Bd*) and *Batrachochytrium salamandrivorans* (*Bsal*) have been described as the etiological agents of the amphibian fungal skin disease chytridiomycosis, which caused the greatest disease-driven loss of amphibian biodiversity ever documented^1–3^. Both chytrid fungi infect the amphibian epidermis, residing intracellularly in the host keratinocytes. Although they show highly similar niche occupancy, the pathogenesis markedly differs between both fungi. *Bd* typically induces epidermal hyperplasia and hyperkeratosis, whereas *Bsal* induces the formation of skin ulcers. Both processes can lead to severe disturbance of skin functioning (including fluid and electrolyte homeostasis and respiration) and subsequent death^4^.

Real time quantitative polymerase chain reaction (RT-qPCR) is a widely used technique for relative gene expression analysis and it has been shown to be a powerful tool for analyzing mRNA expression of pathogens during host infection, uncovering fundamentals of host-pathogen interactions^5^. With the genomes of *Bd* and *Bsal* being fully sequenced, RT-qPCR has already shown its added value in pathogenesis research of chytridiomycosis^5,6^. RT-qPCR results however, largely depend on reference genes which serve as an internal control for standard correction or normalization^7,8^. Reference gene mRNA expression should be stable, meaning that these genes exhibit little variation in their expression within different samples, and their abundance should be in correlation with the total amounts of mRNA in the sample^7^. Selecting suitable reference genes is of utmost importance in order to create reliable results and using more than one reference gene is recommended^9^. Determining reference genes for analyzing fungal gene expression inside host tissue can, however, be challenging. It is difficult to define the amount of pathogen RNA when extracting RNA from an infected tissue. As such, it is tough to determine whether changes in expression are linked to changes in pathogen loads or rather due to instability of the genes.

A thorough screening of reference genes is still lacking in chytrid research. In this study, we therefore examined the stability of 7 reference gene candidates in both *Bd* and *Bsal*. These include translation elongation factor 1-alpha (*TEF1F*), alpha-centractin (*α-centractin*), cysteinyl tRNA synthetase (*Ctsyn1*), glyceraldehyde 3-phosphate dehydrogenase (*GAPDH*), ribosomal protein *R6046* (*R6046*), anthralinate phosphoribosyltransferase (*APRT*) and beta tubulin (*TUB*). Using the statistical software packages ΔCT^10^, GeNorm^11^, NormFinder^12^, BestKeeper^13^ and RefFinder^14^, we assessed the stability of these candidate reference genes during different growth stages of *Bd* and *Bsal*. Subsequently we determined their suitability in host-pathogen research by using an *ex-vivo* approach and finally, we screened their expression profile and stability in tissues of experimentally infected midwife toads (*Alytes obstetricans*) and fire salamanders (*Salamandra salamandra*).

## Results and Discussion

### Cq variation of *Bd* and *Bsal* candidate reference genes during chytrid growth

**Figure 1:**
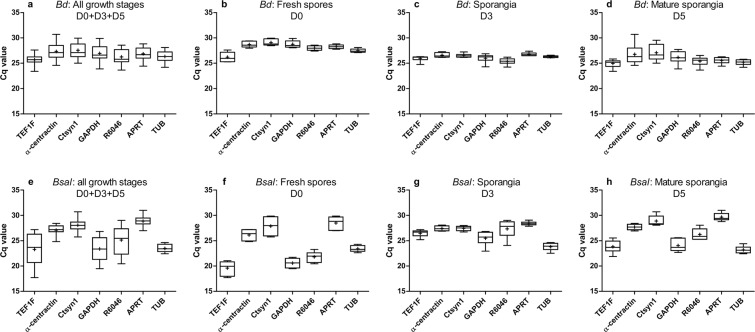
Cq values of 7 reference genes in *Bd* and *Bsal*. Shown is the variation in the mRNA expression (Cq) of *TEF1F*, *α-centractin*, *Ctsyn1*, *GAPDH*, *R6046*, *APRT* and *TUB* in (**a**–**d**) *Bd* and (**e**–**h**) *Bsal* at different growth stages with (**a**,**e**) a combination of all growth stages (D0 + D3 + D5) (n = 18), (**b**,**f**) fresh spores at day 0 (D0) (n = 6), (**c**,**g**) sporangia at day 3 (D3) (n = 6) and (**d**,**h**) a mature culture at day 5 (D5) (n = 6). The whiskers represent the median, the minimum and maximum values, and the first and third quartiles. A plus (+) indicates the mean cq value.

*Bd* and *Bsal* growth and infection of amphibian hosts usually involves multiple developmental stages^2,15,16^. As such, the ideal *in vivo* reference genes should have a constant expression across different developmental stages of the chytrid fungi. Therefore, we investigated the RT-qPCR expression profiles from seven candidate reference genes during *Bd* and *Bsal* growth and determined the Cq values of fresh *Bd* and *Bsal* spores at day 0 (D0), sporangia at day 3 (D3) and mature sporangia at day 5 (D5) (Fig. 1). Different ranges of Cq values were found for the different candidate reference genes, with varying standard deviations (Fig. 1 and Supplementary Tables S1–S2).

For *Bd*, the candidate reference genes showed the least deviation in the spores D0 and sporangia D3 group compared to the mature sporangia at day 5 and a combination of all growth stages, with *TUB* (D0: 27.49 ± 0.37; D3: 26.32 ± 0.19) and *APRT* (D0: 28.27 ± 0.40; D3: 26.77 ± 0.33) showing the lowest standard deviation. In the mature sporangia at day 5, *TUB* (25.19 ± 0.57) and *APRT* (25.56 ± 0.67) also showed the least deviation compared to the other reference genes and when combining all growth stages together, *TUB* (26.33 ± 1.04) and *APRT* (26.87 ± 1.23) were ranked the 2^nd^ and 3^rd^ (Fig. 1a–d and Supplementary Tables S1–S2).

For *Bsal*, the candidate reference genes showed the least deviation in the sporangia group D3 compared to the other groups, with *APRT* (D3: 28.40 ± 0.40) showing the lowest standard deviation. In mature sporangia at day 5, *α-centractin* had the lowest deviation (D5: 27.69 ± 0.61). When examining the spore fraction and a combination of all growth stages, *TUB* was ranked first (D0: 23.41 ± 0.62; D0 + D3 + D5: 23.47 ± 0.72) (Fig. 1e–h and Supplementary Tables S1–S2).

### Expression stability of *Bd* and *Bsal* candidate reference genes during chytrid growth using GeNorm

**Figure 2:**
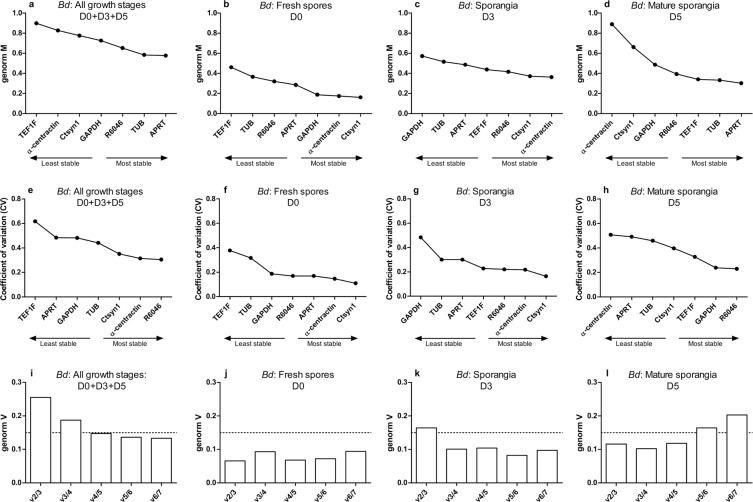
GeNorm stability analysis of *Bd* candidate reference genes. The stability of the genes was assessed during different growth stages with (**a**,**e**,**i**) a combination of all growth stages (D0 + D3 + D5) (n = 18), (**b**,**f**,**j**) fresh spores at day 0 (D0) (n = 6), (**c**,**g**,**k**) sporangia at day 3 (D3) (n = 6) and (**d**,**h**,**l**) a mature culture at day 5 (D5) (n = 6). Genes were ranked based on (**a**–**d**) the GeNorm M and (**e**–**h**) coefficient of variation (CV) value. (**i**–**l**) By pairwise variation (V) analysis, the optimal number of reference genes was determined.

**Figure 3:**
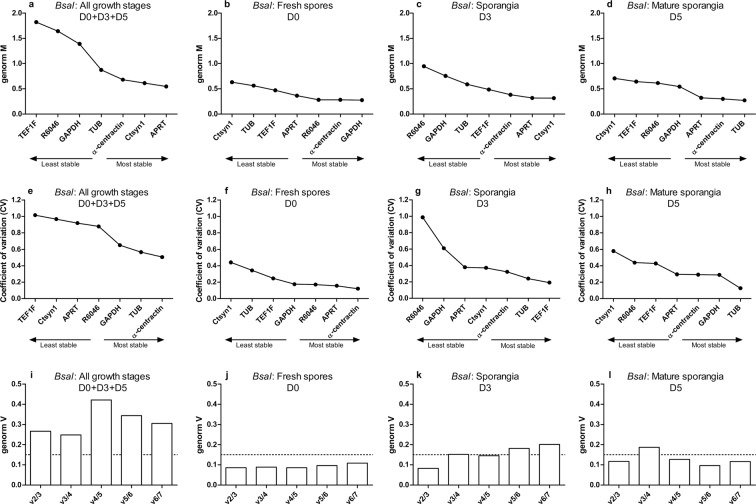
GeNorm stability analysis of *Bsal* candidate reference genes. The stability of the genes was assessed during different growth stages with (**a**,**e**,**i**) a combination of all growth stages (D0 + D3 + D5) (n = 18), (**b**,**f**,**j**) fresh spores at day 0 (D0) (n = 6), (**c**,**g**,**k**) sporangia at day 3 (D3) (n = 6) and (**d**,**h**,**l**) mature culture at day 5 (D5) (n = 6). Genes were ranked based on (**a**–**d**) the GeNorm M and (**e**–**h**) coefficient of variation (CV) value. (**i**–**l**) By pairwise variation (V) analysis, the optimal number of reference genes was determined.

We evaluated the stability of the candidate reference genes during different growth stages of *Bd* and *Bsal* using multiple software packages, assessing different aspects of gene stability. Initially, we analysed the Cq data using GeNorm^11^ (Figs. 2 and 3). GeNorm is based on the expression stability “GeNorm M” value, derived from the average pairwise variation of a potential reference gene set with all other control genes. This means the higher the GeNorm M value, the lower the stability of the reference gene. A coefficient of variation (CV) is also calculated as a relative standard deviation. Ideal reference genes in homogeneous samples, should have an M value ≤ 0.5 and a CV ≤ 0.2, whereas medium reference gene stability (0.5 < M ≤ 1.0) is typically seen in heterogeneous samples^17^. In both *Bd* and *Bsal*, GeNorm M and CV values are the lowest in the spores D0 fraction, reflecting homogeneous samples (Figs. 2b,f and 3b,f).

Using GeNorm analysis of pairwise variation “V” value, the optimal number of reference genes required for normalization was calculated (Figs. 2i–l and 3i–l). This value is an indication for how much difference it makes when using an extra reference gene for normalization. For example, the V2/3 value represents the pairwise variation of two genes compared to that with three genes, the V3/4 value represents the pairwise variation of three genes compared to that with four genes, and so on. If the added value of adding one more is limited (cut-off: V < 0.15), inclusion of an additional reference gene is not necessary. A such, based on GeNorm, RT-qPCR analyses conducted in chytrid spores should contain *Ctsyn1* and *α-centractin* (*Bd*: V2/3 < 0.15), or *GAPDH* and *α-centractin* (*Bsal*: V2/3 < 0.15). When working with only 3 day old sporangia, *α-centractin*, *Ctsyn1* and *R6046* (*Bd*: V3/4 < 0.15) or *Ctsyn1* and *APRT* (*Bsal*: V2/3 < 0.15) should be included. In 5 day old mature sporangia *APRT* and *TUB* (*Bd*: V2/3 < 0.15) or *TUB* and *α-centractin* (*Bsal*: V2/3 < 0.15) provide the best normalization.

In relation to the *in vivo* situation, a combination of all growth stages could be expected in *Bd-* or *Bsal*-infected tissue^2,15,16^. Therefore, we also analysed what reference genes should be included when working with *Bd-* or *Bsal*-infected tissue by taking a combination of all the growth stages into account (spores D0 + sporangia D3 + mature sporangia D5). For *Bd*, the optimal normalization factor could be calculated from *APRT*, *TUB*, *R6046* and *GAPDH*. For *Bsal*, no optimal number of reference targets could be determined as the variability between sequential normalization factors was relatively high (V > 0.15). However, since the use of multiple (non-optimal) reference targets results in more accurate normalization compared to the use of a single non-validated reference target, we recommend the use of at least 3 reference gene candidates or to screen for additional reference genes. Based on the M-value stability, the targets showed the following ranking: *APRT* > *Ctsyn1* > *α-centractin* > *TUB* > *GAPDH* > *R6046* > *TEF1F*.

### Expression stability of *Bd* and *Bsal* candidate reference genes during chytrid growth using ΔCT method, BestKeeper, NormFinder and RefFinder

We further analysed reference gene stability using ΔCT method^10^, BestKeeper^13^, NormFinder^12^ and RefFinder^14^ (Table 1, Supplementary Tables S3–S4). The ΔCT method directly compares relative expression of ‘pairs of genes’ within each sample. The most stable reference genes are associated with the lowest standard deviations of ΔCt when these genes are compared with the other reference genes (Supplementary Tables S3–S4). BestKeeper software evaluates the expression stability of the candidate reference genes based on the standard deviation (SD) of the reference gene Cq values and the coefficient of variation (CV). As a third stability parameter, it calculates the BestKeeper Index from the geometric mean of the remaining reference genes and performs Pearson correlation of each of the reference genes to the BestKeeper index, resulting in a coefficient of correlation (r). Genes with low SD ± CV and high correlation coefficients are the most stable (Supplementary Tables S3–S5). Normally, any studied gene with a SD higher than 1 is considered inconsistent, however, when examining a combination of all developmental stages, including different samples, a low standard deviation for that group was not expected. NormFinder is based on a mathematical model that ranks the candidate reference genes according to their expression stability. This allows the estimation of the overall expression variation of the candidate genes, but also the variations between sample subgroups (e.g. spores day 0 vs sporangia day 3 vs mature sporangia day 5). For each reference gene, a stability value was calculated, taking both intra-and intergroup variations into account (Supplementary Tables S3–S4, Fig. S1). Genes with the lowest stability value are the most stable. In *Bd*, *R6046* and in *Bsal*, *α-centractin*, showed the lowest intra- and intergroup variation and can be defined as the most stably expressed reference genes between and within the different developmental stages (Supplementary Fig. S1). *TEF1F* (*Bd* and *Bsal*) showed the highest intergroup variation and can therefore be defined as the least stable candidate.

**Table 1:** Comprehensive ranking of reference gene stability.

D0 + D3 + D5	rank	GeNorm	RefFinder	D0	rank	GeNorm	RefFinder
		M value	Geomean			M value	Geomean
*Bd*	1	*APRT* (0.58)	*R6046* (1.86)	*Bd*	1	*Ctsyn1* (0.16)	*Ctsyn1* (1.50)
	2	*TUB* (0.58)	*APRT* (2.63)		2	*α-centractin* (0.18)	*α-centractin* (2.00)
	3	*R6046* (0.65)	*TUB* (2.66)		3	*GAPDH* (0.19)	*APRT* (3.36)
	4	*GAPDH* (0.73)	*GAPDH* (3.13)		4	*APRT* (0.29)	*R6046* (3.41)
	5	*Ctsyn1* (0.78)	*Ctsyn1* (4.21)		5	*R6046* (0.32)	*TUB* (3.83)
	6	*α-centractin* (0.83)	*TEF1F* (4.30)		6	*TUB* (0.37)	*GAPDH* (4.61)
	7	*TEF1F* (0.90)	*α-centractin* (5.73)		7	*TEF1F* (0.46)	*TEF1F* (7.00)
*Bsal*	1	*APRT* (0.55)	*α-centractin* (1.57)	*Bsal*	1	*GAPDH* (0.27)	*α-centractin* (1.86)
	2	*Ctsyn1* (0.61)	*APRT* (2.21)		2	*α-centractin* (0.28)	*R6046* (1.86)
	3	*α-centractin* (0.68)	*TUB* (2.63)		3	*R6046* (0.28)	*GAPDH* (2.21)
	4	*TUB* (0.87)	*Ctsyn1* (3.16)		4	*APRT* (0.33)	*TUB* (3.83)
	5	*GAPDH* (1.39)	*GAPDH* (3.50)		5	*TEF1F* (0.39)	*APRT* (3.94)
	6	*R6046* (1.64)	*R6046* (6.00)		6	*TUB* (0.46)	*TEF1F* (5.23)
	7	*TEF1F* (1.82)	*TEF1F* (7.00)		7	*Ctsyn1* (0.56)	*Ctsyn1* (7.00)
**D3**	**rank**	**GeNorm**	**RefFinder**	**D5**	**rank**	**GeNorm**	**RefFinder**
		**M value**	**Geomean**			**M value**	**Geomean**
*Bd*	1	*α-centractin* (0.36)	*Ctsyn1* (1.32)	*Bd*	1	*APRT* (0.30)	*TUB* (2.24)
	2	*Ctsyn1* (0.37)	*α-centractin* (2.11)		2	*TUB* (0.33)	*R6046* (2.38)
	3	*R6046* (0.42)	*TEF1F* (3.46)		3	*TEF1F* (0.34)	*APRT* (2.38)
	4	*TEF1F* (0.44)	*TUB* (3.66)		4	*R6046* (0.39)	*TEF1F* (2.71)
	5	*APRT* (0.49)	*R6046* (4.12)		5	*GAPDH* (0.49)	*GAPDH* (2.94)
	6	*TUB* (0.52)	*APRT* (4.16)		6	*Ctsyn1* (0.66)	*Ctsyn1* (6.00)
	7	*GAPDH* (0.57)	*GAPDH* (7.00)		7	*α-centractin* (0.89)	*α-centractin* (7.00)
*Bsal*	1	*Ctsyn1* (0.22)	*TEF1F* (2.00)	*Bsal*	1	*TUB* (0.27)	*TUB* (1.19)
	2	*APRT* (0.23)	*APRT* (2.11)		2	*α-centractin* (0.30)	*α-centractin* (1.57)
	3	*α-centractin* (0.24)	*Ctsyn1* (2.51)		3	*APRT* (0.32)	*APRT* (2.71)
	4	*TEF1F* (0.43)	*α-centractin* (2.71)		4	*GAPDH* (0.54)	*R6046* (4.47)
	5	*TUB* (0.56)	*TUB* (3.50)		5	*R6046* (0.61)	*GAPDH* (5.14)
	6	*GAPDH* (0.75)	*GAPDH* (6.00)		6	*TEF1F* (0.64)	*TEF1F* (6.00)
	7	*R6046* (0.95)	*R6046* (7.00)		7	*Ctsyn1* (0.71)	*Ctsyn1* (6.09)

GeNorm and RefFinder were used to determine reference gene stability in *Bd* and *Bsal* spores day 0 (D0) (n = 6), sporangia day 3 (D3) (n = 6), mature sporangia day 5 (D5) (n = 6) and a combination of all life stages (D0 + D3 + D5) (n = 18). Candidate genes were ranked from most stable (1) to least stable (7).

Depending on the algorithm used, varying rankings for *Bd* and *Bsal* candidate reference genes were observed (Supplementary Tables S3–S4). Therefore, we applied RefFinder, a tool developed to generate a comprehensive ranking of the most stable reference genes by integrating data obtained by ΔCT, GeNorm, NormFinder and BestKeeper. Using the ranking from each program, RefFinder assigns an appropriate weight to a candidate reference gene and it calculates the geometric mean of their weights for the overall ranking^14^. The analysis of the comprehensive ranking revealed that indeed *R6046*, *APRT*, *TUB* and GAPDH are the most stably expressed genes throughout all the developmental stages of *Bd*. For *Bsal*, slightly deviant results were obtained compared to the results obtained by GeNorm analysis alone, as *α-centractin*, *APRT* and *TUB* instead of *Ctsyn1*, were shown to be most stably expressed. Also, in the separate growth stages, slight differences were observed between the GeNorm and overall RefFinder ranking (Table 1).

### *Ex vivo* expression analysis of chytrid reference gene candidates in contact with skin tissue

In order to determine whether the targets are suitable as *in vivo* reference genes, we first analysed whether they stay stably expressed if the fungi come in contact with host skin using an *ex vivo* approach. Based on the results described above and with relation to the *in vivo* situation where infected skin tissue comprises different developmental stages of the fungus, we evaluated the stability of *GAPDH*, *TUB*, *R6046* and *APRT* for *Bd* when a standardized amount of the fungus encounters host tissue. In *Bsal*, *Ctsyn1* and *APRT* were shown to be among the most stably expressed genes in a combination of all growth stages (Fig. 3a), however with an average Cq value of 28.07 ± 1.30 and 28.86 ± 1.02 (Fig. 1e and Supplementary Table S2). This is the highest of all tested targets, making these candidates less suitable as *in vivo* reference genes. *TEF1F* was shown to be the least stably expressed gene of all targets tested, with the highest intergroup variation (Table 1 and Supplementary Fig. 1). Therefore, we analysed the stability of *GAPDH*, *TUB*, *R6046* and *α-centractin* if host tissue is added to *Bsal*.

**Figure 4:**
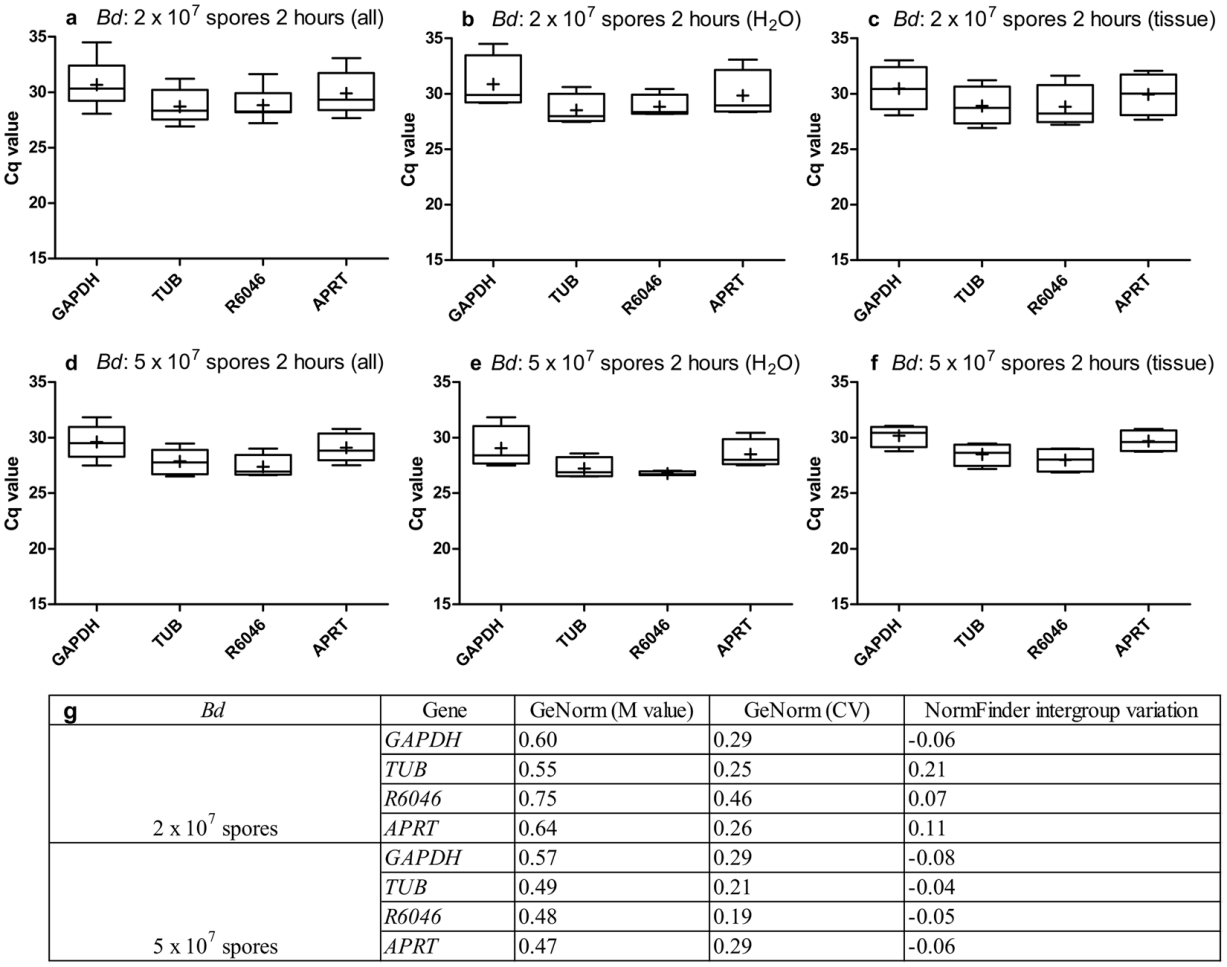
*Ex vivo* expression analysis of *Bd* reference gene candidates. Shown is the variation in the mRNA expression (Cq) of *GAPDH*, *TUB*, *R6046* and *APRT* in (**a**–**c**) 2 × 10^7^ *Bd* spores and (**d**–**f**) 5 × 10^7^ *Bd* spores that were incubated for 2 hours at 20 °C (**c**,**f**) with or (**b**,**e**) without skin tissue of midwife toads, or (**a**,**d**) a combination of both. Every condition was tested in fourfold. The whiskers represent the median, the minimum and maximum values, and the first and third quartiles. A plus (+) indicates the mean Cq value. (**g**) GeNorm stability analysis providing a GeNorm M and coefficient of variation (CV) value, combined with NormFinder intergroup variation analysis.

**Figure 5:**
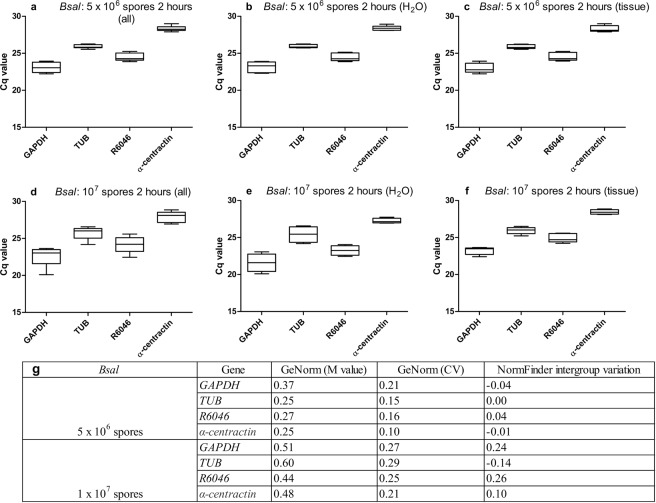
*Ex vivo* expression analysis of *Bsal* reference gene candidates. Shown is the variation in the mRNA expression (Cq) of *GAPDH*, *TUB*, *R6046* and *α-centractin* in (**a**–**c**) 5 × 10^6^ *Bsal* spores and (**d**–**f**) 1 × 10^7^ *Bsal* spores that were incubated for 2 hours at 15 °C (**c**,**f**) with or (**b**,**e**) without skin tissue of fire salamanders, or (**a**,**d**) a combination of both. Every condition was tested in fivefold. The whiskers represent the median, the minimum and maximum values, and the first and third quartiles. A plus (+) indicates the mean Cq value. (**G**) GeNorm stability analysis providing a GeNorm M and coefficient of variation (CV) value, combined with NormFinder intergroup variation analysis.

In the groups without skin tissue, only fungal RNA was isolated, whereas in the groups with skin tissue, a combination of fungal and host RNA was isolated. This led to a small variation in Cq values between the groups “with” (Figs. 4c,f and 5c,f) and “without” (Figs. 4b,e and 5b,e) skin tissue (Supplementary Table S1). However, this happened in a standardized way, allowing us to check the stability of the reference genes and whether or not this changed when the spores came in contact with skin. We focused on the results obtained by GeNorm analysis (M and CV value) and the intergroup variation obtained by NormFinder.

For *Bd* at a concentration of 2 × 10^7^ spores, *GAPDH*, *TUB*, *R6046* and *APRT* show a medium reference gene stability (0.5 ≤ M ≤ 1.0). When increasing the fungal load and, as a result the proportion of fungal RNA within the mix of host and fungal RNA, *TUB*, *R6046* and *APRT* even showed a high reference gene stability (M ≤ 0.5) (Fig. 4g). For *Bsal* at a concentration of 5 × 10^6^ spores, a high reference gene stability (M ≤ 0.5) was observed for *GAPDH*, *TUB*, *R6046* and *α-centractin* (Fig. 5g), as well as for *R6046* and *α-centractin* at a *Bsal* concentration of 10^7^ spores. Despite the fact that we were dealing with mixed host and fungal RNA vs only fungal RNA, NormFinder analysis showed a relatively small intergroup variation for both *Bd* and *Bsal*. This indicates that these reference genes stay stably expressed when *Bd* and *Bsal* come in contact with host tissue and that they could serve as normalization factors for chytrid-infected skin samples.

### Expression profile and stability of chytrid candidate reference genes in experimentally infected amphibians

Based on the above described results, *GAPDH*, *TUB*, *R6046* and *APRT* were proposed as candidate reference genes in *Bd* and *GAPDH*, *TUB*, *R6046 and α-centractin* in *Bsal* for *in vivo* normalization. We now analysed whether they indeed could be validated as *in vivo* reference genes by examining their expression stability in skin tissue of experimentally *Bd*-infected midwife toads and *Bsal*-infected fire salamanders and by assessing the expression of target genes.

**Figure 6:**
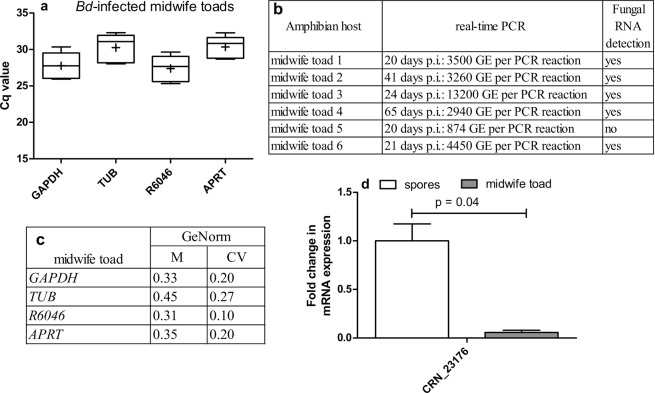
*In vivo* expression profile and stability of *Bd* candidate reference genes. Shown is (**a**) the variation in the mRNA expression (Cq) of *GAPDH*, *TUB*, *R6046* and *APRT* in skin tissue (thigh) of experimentally *Bd*-infected midwife toads (n = 6). The whiskers represent the median, the minimum and maximum values, and the first and third quartiles. A plus (+) indicates the mean Cq value. (**b**) At euthanasia, different fungal loads were detected in the skin tissues. (**c**) GeNorm stability analysis providing a GeNorm M and coefficient of variation (CV) value. (**d**) *CRN_23176* mean fold change in mRNA expression profiles. The data shows the normalized target gene quantity in skin tissue from *Bd*-infected midwife toads, relative to freshly collected spores which is considered 1. The results are presented as means + standard error of the mean (SEM) with significant differences shown by the P value.

**Figure 7:**
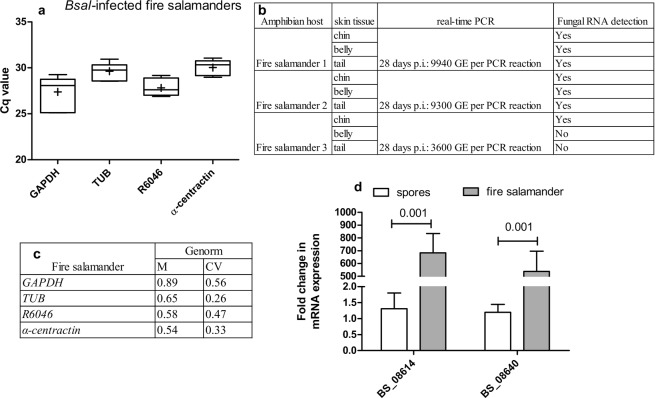
*In vivo* expression profile and stability of *Bsal* candidate reference genes. Shown is (**a**) the variation in the mRNA expression (Cq) of *GAPDH*, *TUB*, *R6046* and *α-centractin* in skin (chin, belly and tail) tissue of experimentally *Bsal*-infected fire salamanders (n = 3). The whiskers represent the median, the minimum and maximum values, and the first and third quartiles. A plus (+) indicates the mean Cq value. (**b**) At euthanasia, different fungal loads were detected in the skin tissues. (**c**) GeNorm stability analysis providing a GeNorm M and coefficient of variation (CV) value. (**d**) *BS_08640* and *BS_08614* mean fold change in mRNA expression profiles. The data shows the normalized target gene quantities in skin tissue from *Bsal*-infected *Salamandra salamandra*, relative to freshly collected spores which is considered 1. The results are presented as means + SEM with significant differences shown by the P value.

The chytrid loads differed between the different animals. As such, differences in Cq values were expected as a result of differences in pathogen load inside host tissue (Figs. 6a,b and 7a,b). When analyzing intracellular fungal pathogens, there is no straightforward method for separating fungal and host RNA, highlighting the need of good reference genes for the normalization of RT-qPCR data from mixed host-fungi RNA samples. When assessing the stability of *Bd* candidate reference genes in infected skin tissue of midwife toads using GeNorm, M values ≤ 0.5 (*GAPDH*, *R6046*, *APRT* and *TUB*) and CV values ≤ 0.2 (*GAPDH*, *R6046*, *APRT*) were observed, indicating high reference gene stability (Fig. 6c). In skin tissue of *Bsal*-infected fire salamanders a medium reference gene stability was observed (M ≤ 1.0) (Fig. 7c).

To examine the utility of the candidate reference genes, the expression of target genes *CRN_23176* (*Bd*), *BS_08640* (*Bsal*) and *BS_08614* (*Bsal*) was determined using a combination of *GAPDH*, *TUB*, *R6046* and *APRT* for *Bd* and *GAPDH*, *TUB*, *R6046* and *α-centractin* for *Bsal* as normalizers. According to Farrer *et al*. (2017), the Crinkler and Necrosis gene *CRN_23176* is highly expressed during early interaction of *Bd* with the host, whereas it is less expressed during advanced *Bd* infection of *Tylototriton wenxianensis*^5^. We noticed a significant downregulation of *CRN_23176* (p < 0.05) in skin tissue of *Bd* positive midwife toads compared to spores. This confirms the results by Farrer *et al*. (2017) in midwife toads as a host and validates the use of *GAPDH*, *TUB*, *R6046* and *APRT* as *Bd* reference genes for *in vivo* screening of target genes (Fig. 6d)^5^. *BS_08640* and *BS_08614* are two target genes with an unknown function, however, using RNA-seq analysis they were shown to be highly expressed in the skin of *Bsal*-infected *Tylototriton wenxianensis* animals^5^. When using *GAPDH*, *TUB*, *R6046* and *α-centractin* as reference genes, a significant (p < 0.05) upregulation of both genes of more than 500 times was observed in *Bsal*-infected skin tissue of fire salamanders, compared to expression levels in spores (Fig. 7d). These results are in line with data described by Farrer *et al*. (2017), highlighting the utility of the *Bsal* reference gene candidates and the importance of *BS_08640* and *BS_08614* during infection of the host with *Bsal*^5^.

All too often, only one reference gene or even non-validated reference genes are used in an RT-qPCR setup which can lead to misleading results^18^. A careful selection of thoroughly validated reference genes prior to performing qPCR experiments is therefore recommended. However, the ideal set of reference genes does not exist, i.e. reference genes whose expression is constant across all cells and tissues. *Bd* and *Bsal* are fungal organisms that show changes in expression among isolates^19–21^, by serial passages in artificial culture medium^22^ and depending on the amphibian host^22^. As such, it should be taken into account that these factors and possibly others (e.g. chemical treatment, type of tissue or *in vivo*-infection conditions) could also affect the stability of the suggested reference genes. Therefore, our results serve as a guide during the *Bd* and *Bsal* reference gene hunt but depending on the experimental setup researchers should carefully think out the best normalization strategy.

Summarized, we investigated the stability of suitable reference genes during the development of *Bd* and *Bsal* and depending on the growth stage, different combinations of candidate reference genes should be used (Supplementary Table S6). We further focused on defining reference genes that can be used for mRNA expression analysis of infected host tissue and we propose that the optimal *in vivo* normalization factor can be calculated using *GAPDH*, *TUB*, *R6046* and *APRT* for *Bd* and *GAPDH*, *TUB*, *R6046* and *α-centractin* for *Bsal* (Supplementary Table S6). This study will enhance the accuracy of future RT-qPCR analyses of *in vitro*, *ex vivo* and *in vivo Bd* and *Bsal* studies.

## Methods

### Zoospore isolation

*Bd* and *Bsal* spores were collected from mature cultures in sterile distilled water. In order to reduce the percentage of mature cells, the water containing the zoospores was passed over a sterile mesh filter with pore size 10 µm (Pluristrainer, PluriSelect). The flow through was used as the zoospore fraction (>90% purity).

### *In vitro Bd* and *Bsal* culture conditions

The zoospore fraction (3 ml) was seeded in 6-well plates at a density of 2.5 × 10^5^ spores/ml TGhL (1.6% tryptone, 0.4% gelatin hydrolysate and 0.2% lactose in H_2_O) for *Bd* and 7.5 × 10^5^ spores/ml TGhL for *Bsal*. The spores were incubated during 5 days at 20 °C or 15 °C, respectively. RNA was extracted from the *Bd* and *Bsal* cultures during different growth stages, namely at day 0 (D0: fresh spores), day 3 (D3: sporangia) and day 5 (D5: mature sporangia containing zoospores). Every condition was tested in sixfold.

### *Ex vivo Bd* and *Bsal* infection experiments

Testing whether the reference gene candidates stay stable inside infected host tissue, can pose some problems. When extracting RNA from chytrid-infected tissue, there is no straightforward way of determining the amount of fungal RNA inside the pool of host and fungal RNA. Therefore, changes in reference gene expression could be the result of instability of these genes, or from an increase in pathogen load in host tissue. In order to determine whether candidate reference genes indeed stay stable when they come in contact with host tissue, we performed an *ex vivo* experiment. A standardized amount of spores (*Bd*: 2 × 10^7^ and 5 × 10^7^; *Bsal*: 5 × 10^6^ and 1 × 10^7^) were incubated for 2 hours in H_2_O with skin tissue of a chytrid-susceptible amphibian (*Bd*: midwife toad; *Bsal*: fire salamander), collected with a skin biopsy punch (6 mm)^2,23–26^. As a control, we included *Bd* and *Bsal* zoospores that were incubated without tissue. All samples were incubated for 2 hours at 20 °C or 15 °C, respectively, after which RNA was extracted. Every condition was tested in fourfold (*Bd*) or fivefold (*Bsal*).

### *In vivo Bd* and *Bsal* infection experiments

This study was carried out in strict accordance with the recommendation in the European Convention for the Protection of Vertebrate Animals used for Experimental and other Scientific Purposes. Animal experiments were performed with the approval of the ethical committee of the Faculty of Veterinary Medicine (Ghent University 2016/120 and 2016/55). All animals used were clinically healthy and free of *Bd* and *Bsal* as assessed by sampling the skin using cotton-tipped swabs and subsequent performing qPCR. All animals were acclimatized for 1 week before the onset of the experiments. The animals were housed individually at 15 ± 1 °C on moist tissue, with access to a hiding place and they were fed daily.

In a first experiment, six captive bred midwife toads were exposed to 1 ml of 10^6^ *Bd* spores per ml water for 24 h at 15 ± 1 °C. Animals were followed up by clinical examination and the infection load was followed up weekly by taking swabs on which we performed qPCR. The animals were euthanized when clinical symptoms were observed (e.g. lethargy, loss of appetite, weight reduction). A part of the skin (10 mg: thigh) was stored in RNA later for 24 h and subsequently stored at −70 °C. Skin samples for histopathology were stored in formalin.

In a second experiment, three captive bred fire salamanders were exposed to 1 ml of 10^3^ *Bsal* spores per ml water for 24 hours at 15 ± 1 °C. The animals were swabbed and euthanized at day 28 post-infection. A part of the skin (10 mg: belly, chin and tail) was stored in RNA later for 24 h and subsequently stored at −70 °C. Skin samples for histopathology were stored in formalin.

### RNA extraction and cDNA synthesis

Using the RNeasy mini kit (Qiagen), total RNA was isolated from *in vitro Bd* and *Bsal* cultures, *ex vivo Bd* and *Bsal*-infected skin samples and *in vivo Bd* and *Bsal*-infected skin samples. The RNA concentration was measured by absorbance at 260 nm using a nanodrop spectrophotometer and the quality of the RNA samples was checked using an Experion RNA StdSens Analysis kit (Bio-Rad). Total RNA (500 ng) was reverse transcribed to cDNA with the iScript cDNA synthesis kit (Bio-Rad) and cDNA was stored at −20 °C until further use.

### Reference genes and primer design

*Bd* and *Bsal* primers for *α-centractin*, *R6046*, *TEF1F* and *GAPDH* were used from previously published data^5^. *Bd* and *Bsal* primers for *APRT*, *Ctsyn1* and *TUB* were designed using Primer3plus (Supplementary Table S7). The specificity of each primer set was checked by nucleotide blast and by performing a standard PCR (40 cycles) on *Bd* and *Bsal* cDNA (diluted 1:5), followed by gel electrophoresis. The PCR products were checked on an agarose gel (1.5%) and single band amplification was confirmed (Supplementary Figs. S2–S3). Primer efficiency was evaluated by performing real-time quantitative PCR reactions on serial dilutions of a cDNA mix from the *in vitro* samples (1:5, 1:25, 1:125; 1:625). For every standard curve, we assessed the amplicon efficiency (E), correlation coefficients (R^2^) and slope (Supplementary Table S7). Water and no-template controls were used as negative controls for each primer set. Melting curves were analysed and for all primer pairs a single peak was detected (Supplementary Figs. S4-S5).

### RT-qPCR analysis and data analysis

Real-time quantitative PCR reactions were run in duplicate and the reactions were performed in 10 μl volumes using the iQ SYBR Green Supermix (Bio-Rad) and 1 µl 1/5 diluted cDNA. The experimental protocol for PCR (40 cycles) was performed on a CFX384™ RT-qPCR System with a C1000 Thermal Cycler (Bio-Rad, Hercules). The results were analysed using the Bio-Rad CFX manager 3.1. Quantification cycle (Cq) values were obtained using auto baseline settings and they were applied per primer set. The threshold for maximum Cq difference between the technical replicates was set to 0.5. We used different statistical algorithms to assess the stability of the candidate reference genes. Therefore, the RT-qPCR data obtained were exported into an Excel datasheet and the raw Cq values (Supplementary Table S1) were used directly for analysis in Qbase (GeNorm)^17^, BestKeeper^13^ and the ΔCT method^10^. For NormFinder analysis^12^, the raw Cq values were converted into relative quantities using the formula Q = E^−ΔCq^, with E = amplification efficiency and ΔCq = the corresponding Cq value – minimum Cq. The RefFinder tool integrates the data obtained from GeNorm, BestKeeper, ΔCT and NormFinder analysis and calculates a comprehensive ranking order^14^. All the software packages were used according to the manufacturer’s instructions.

### Validation of *in vivo* reference genes by expression analysis of target genes

The *Bd* Crinkler and Necrosis gene *CRN_23176* and *Bsal in vivo* genes *BS_08640* and *BS_08614*, were used as target genes to analyse the usefulness of the selected candidate reference genes^5^. The results are shown as fold changes of mRNA expression, which were calculated based on the CNRQ values obtained in qBase. Statistics were performed using SPSS version 25 (SPSS Inc., Chicago, IL, USA), by performing a non-parametric Kruskal-Wallis analysis on the CNRQ values, with significance set to p < 0.05.

## Supplementary information

Supplementary information

## Data Availability

All data generated or analysed during this study are included in this published article and its Supplementary Information Files.
