# Supplementary material for: Reference gene screening of Batrachochytrium dendrobatidis and Batrachochytrium salamandrivorans for quantitative real-time PCR studies
Source: Sci Rep. 2019 Dec 6;9:18534. doi: 10.1038/s41598-019-54582-4 (PMC6898426; doi:10.1038/s41598-019-54582-4)
Supplement: Supplementary file 1 — Supplementary information [file 41598_2019_54582_MOESM1_ESM.pdf]

Reference gene screening of *Batrachochytrium dendrobatidis* and *Batrachochytrium salamandrivorans* for quantitative real-time PCR studies

Elin Verbrugghe[1], Frank Pasmans[1], An Martel[1]

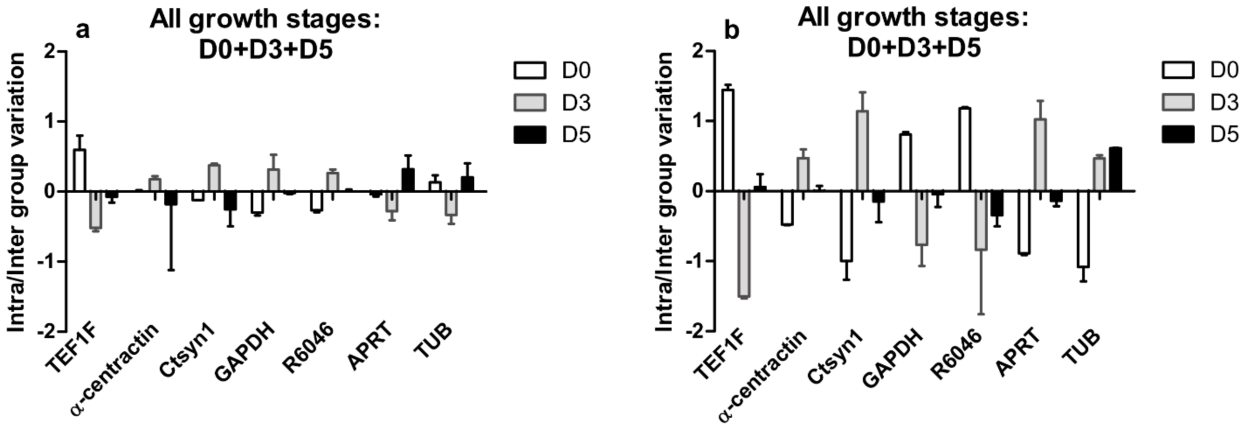

Supplementary Figure S1: Intra-and intergroup variation as estimated by NormFinder. The columns represent the intergroup variation and the error bars indicate the intragroup variations of **(a)** *Bd* and **(b)** *Bsal* candidate reference genes between spores day 0 (D0) (n = 6), sporangia day 3 (D3) (n = 6) and mature sporangia day 5 (D5) (n = 6). The most stable gene would be that having as small error bars as possible and with an intergroup variation close to zero.

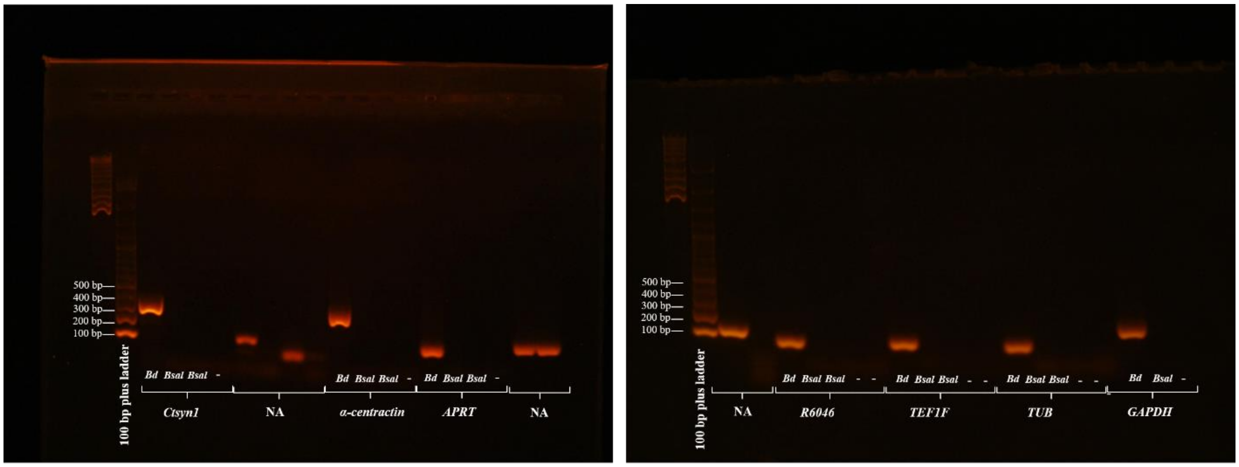

Supplementary Figure S2: Agarose gel electrophoresis of *Bd* PCR products. Shown are full-length gels showing the PCR products of the tested *Bd* reference genes (*Ctsyn1*, *α-centractin*, *APRT*, *R6046*, *TEF1F*, *TUB* and *GAPDH*) using *Bd* and *Bsal* cDNA (1/5 diluted). For every primer pair a water template control was included (-). Ladder = generuler 100 bp plus (Thermo Scientific). NA = not applicable for this article.

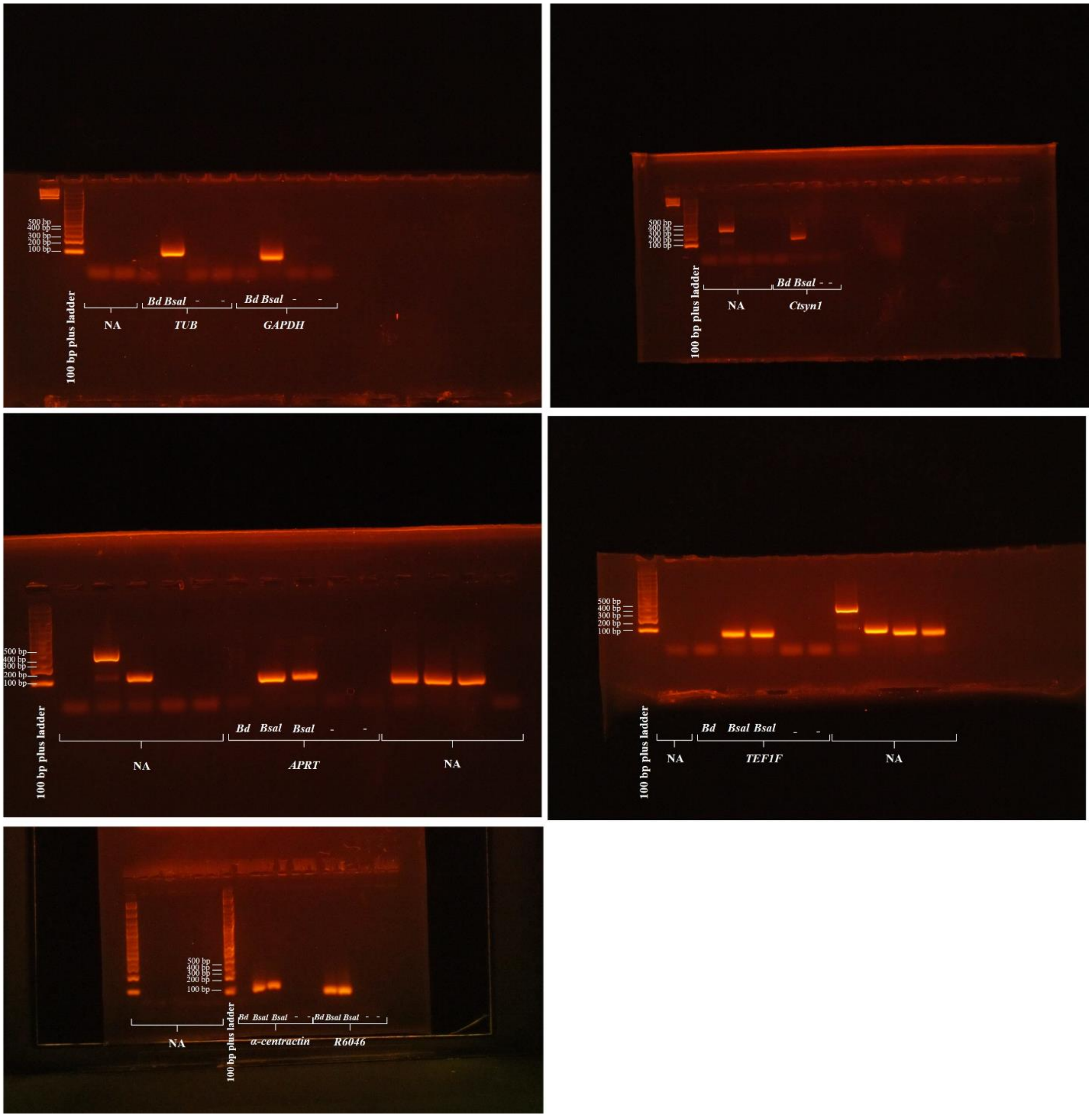

Supplementary Figure S3: Agarose gel electrophoresis of *Bsal* PCR products. Shown are full-length gels showing the PCR products of the tested *Bsal* reference genes (*Ctsyn1*, *α-centractin*, *APRT*, *R6046*, *TEF1F*, *TUB* and *GAPDH*) using *Bd* and *Bsal* cDNA (1/5 diluted). For every primer pair a water template control was included (-). Ladder = generuler 100 bp plus (Thermo Scientific). NA = not applicable for this article.

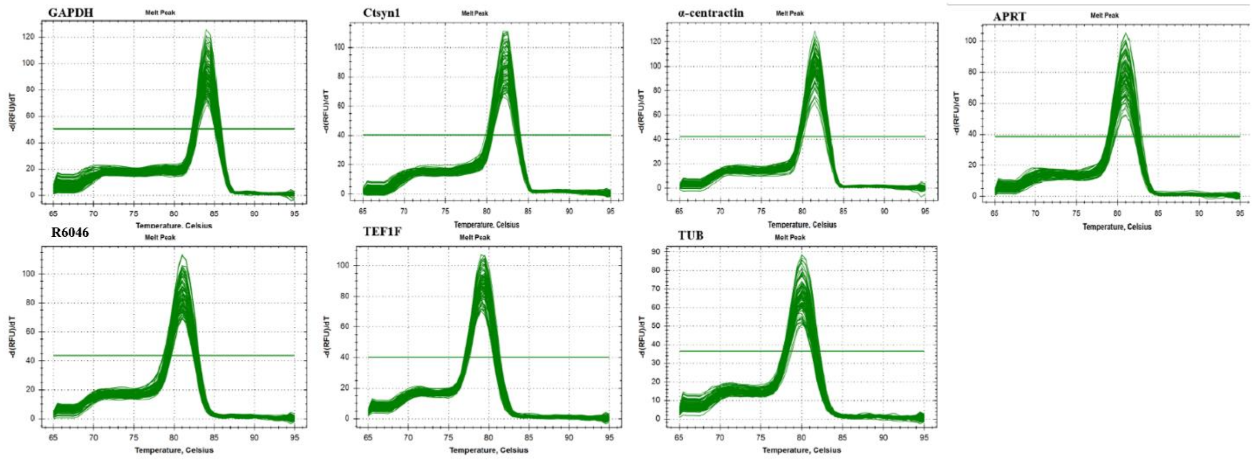

Supplementary Figure S4: Melting curve analysis of *Bd* amplicons. Melting curves of 7 *Bd* genes (*α-centractin*, *Ctsyn1*, *APRT*, *R6046*, *TEF*, *TUB*, *GAPDH*) showing single peaks.

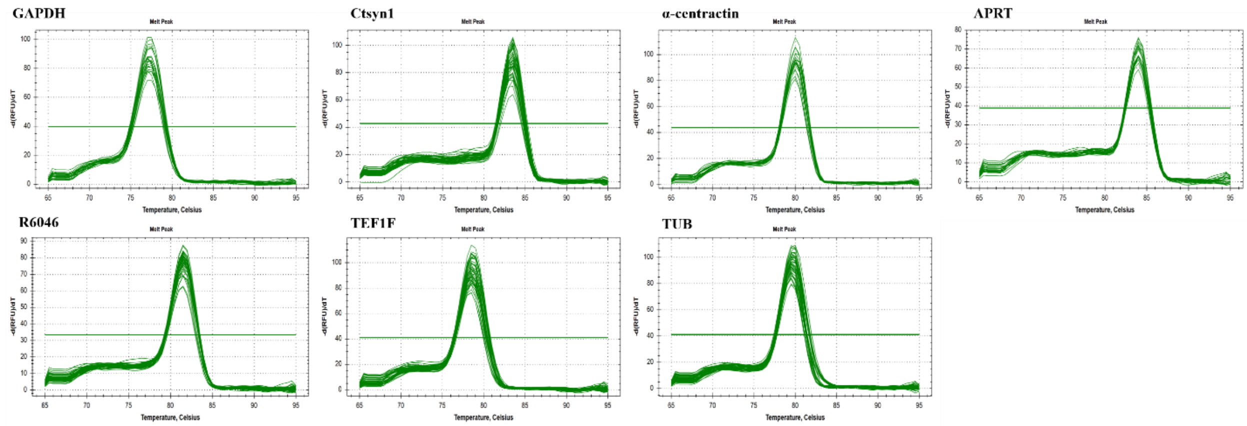

Supplementary Figure S5: Melting curve analysis of *Bsal* amplicons. Melting curves of 7 *Bsal* genes (*α-centractin*, *Ctsyn1*, *APRT*, *R6046*, *TEF*, *TUB*, *GAPDH*) showing single peaks.

Supplementary Table S1: Cq values of all individual samples.

		Bd	*α-centractin*	*Ctsyn1*	*APRT*	*R6046*	*TEF1F*	*TUB*	*GAPDH*
experiment 1	1	Spores D0	29.41	29.96	28.83	28.40	27.61	27.49	29.91
	2		28.66	28.82	27.96	27.41	25.27	27.11	28.64
	3		27.98	28.47	27.76	27.63	26.30	27.31	28.06
	4		28.47	28.80	28.10	27.88	25.62	27.22	28.35
	5		28.29	28.63	28.42	28.06	25.29	27.71	28.23
	6		29.37	29.80	28.56	28.57	27.05	28.11	29.26
	1	Sporangia D3	26.22	26.14	26.90	24.24	24.76	26.41	24.29
	2		26.63	26.55	26.71	25.29	26.04	26.58	26.05
	3		26.21	26.21	26.68	25.31	26.18	26.08	26.23
	4		26.15	26.38	26.47	25.46	26.19	26.39	26.36
	5		27.08	26.58	26.49	26.20	26.24	26.31	26.87
	6		27.29	27.24	27.37	25.76	26.30	26.13	26.25
	1	Mature sporangia D5	25.70	26.32	25.31	25.59	24.70	25.36	25.98
	2		25.52	26.12	25.86	25.19	25.28	25.15	26.08
	3		26.90	26.96	25.40	25.61	25.15	24.98	26.07
	4		24.58	25.02	24.42	23.66	23.41	24.19	23.87
	5		30.70	29.56	26.07	26.54	25.84	25.79	27.71
	6		27.12	28.48	26.28	25.83	25.35	25.65	27.12
experiment 2	1	2 x 10^7 spores 2 hours H_2O	29.25	30.53	28.56	28.42	27.62	27.76	29.38
	2		34.15	31.69	33.08	30.43	29.14	30.61	34.51
	3		32.69	30.52	29.36	28.28	27.98	28.22	30.39
	4		30.24	30.41	28.35	28.18	27.84	27.48	29.18
	1	2 x 10^7 spores 2 hours H_2O + tissue midwife toad	30.13	31.35	30.75	28.24	28.01	29.00	30.59
	2		29.65	30.72	29.30	28.21	27.06	28.48	30.26
	3		32.50	33.36	32.07	31.65	30.82	31.22	33.02
	4		27.74	28.08	27.67	27.21	26.23	26.93	28.06
	1	5 x 10^7 spores 2 hours H_2O	26.93	27.49	27.51	26.62	24.54	26.55	27.49
	2		30.41	33.05	30.42	26.62	27.33	28.58	31.84
	3		25.43	26.91	27.89	27.02	24.65	27.21	28.15
	4		27.55	28.14	28.14	26.76	24.17	26.51	28.68
	1	5 x 10^7 spores 2 hours H_2O + tissue midwife toad	30.40	31.22	30.25	29.01	27.47	29.48	31.06
	2		30.28	31.53	30.80	27.21	25.48	29.00	30.65
	3		28.09	28.22	28.73	26.86	25.24	27.18	28.78
	4		29.61	29.58	28.96	28.85	28.68	28.29	30.22
experiment 3	1	spores	29.41	29.96	28.83	28.40	27.61	27.49	29.91
	2		28.66	28.82	27.96	27.41	25.27	27.11	28.64
	3		27.98	28.47	27.76	27.63	26.30	27.31	28.06
	4		28.47	28.80	28.10	27.88	25.62	27.22	28.35
	5		28.29	28.63	28.42	28.06	25.29	27.71	28.23

	6		29.37	29.80	28.56	28.57	27.05	28.11	29.26
	1	*Bd*-infected skin of midwife toads	30.62	32.63	30.98	28.48	27.13	31.57	28.70
	2		32.54	33.08	32.30	29.64	29.39	32.31	30.34
	3		28.57	28.40	28.67	25.32	24.77	28.31	25.93
	4		27.87	29.59	28.94	25.85	25.65	28.04	26.16
	5		ND	ND	ND	ND	ND	ND	ND
	6		30.60	32.05	30.83	27.67	27.22	31.07	27.75
	Bsal		*α-centractin*	*Ctsyn1*	*APRT*	*R6046*	*TEF1F*	*TUB*	*GAPDH*
experiment 1	1	Spores D0	26.36	27.88	28.58	22.07	20.33	23.33	20.79
	2		26.39	28.10	29.06	21.85	19.56	23.25	20.42
	3		27.21	29.70	29.61	22.40	20.76	23.97	21.43
	4		27.17	29.88	29.85	23.26	21.04	24.28	21.73
	5		24.82	25.99	27.01	20.44	17.71	22.62	19.63
	6		24.95	25.74	26.97	20.74	18.01	22.99	19.49
	1	Sporangia D3	27.2	27.04	28.18	28.54	26.19	23.51	25.59
	2		27.9	27.77	28.53	26.69	26.83	23.43	25.12
	3		26.87	26.73	27.86	27.12	26.62	24.35	25.86
	4		26.95	27.43	28.42	24.08	25.21	22.52	22.94
	5		27.53	27.63	28.32	29.02	26.77	24.63	26.82
	6		28.09	28.00	29.06	28.63	27.18	24.43	26.63
	1	Mature sporangia D5	27.36	28.32	29.37	26.07	23.94	22.89	23.53
	2		28.17	28.08	29.42	27.09	24.70	23.49	25.59
	3		26.94	28.24	28.78	25.26	21.87	22.41	22.67
	4		27.18	28.36	29.24	25.64	23.45	22.63	23.17
	5		28.06	29.59	30.19	25.25	23.31	23.35	23.84
	6		28.43	30.73	30.99	28.07	25.53	24.40	25.48
experiment 2	1	5 x 10^6 spores 2 hours H_2O	28.39	29.44	30.74	24.96	21.55	26.14	23.31
	2		28.38	29.62	30.04	24.13	21.76	25.78	23.91
	3		28.09	28.17	30.58	24.26	21.38	25.84	22.31
	4		28.12	30.16	31.5	23.87	21.75	25.73	22.44
	5		28.94	30.56	31.94	25.15	22.3	26.25	23.76
	1	5 x 10^6 spores 2 hours H_2O + tissue fire salamander	28.47	29.33	30.88	25.01	21.78	26.13	23.38
	2		28.09	29.52	30.38	24.21	21.63	25.8	22.78
	3		27.9	28.83	30.57	24.27	21.29	25.76	22.68
	4		28.11	30.31	30.94	23.96	21.72	25.55	22.23
	5		29	30.56	30.69	25.26	22.5	26.24	23.93
	1	10^7 spores 2 hours H_2O	27.05	27.16	29.03	23.47	21.14	24.88	21.4
	2		27.74	27.41	28.52	24.04	21.95	26.57	23.07
	3		26.95	28.99	29.69	22.46	20.77	24.18	20.09
	4		27.23	27.64	28.5	23.03	20.89	26.04	21.79

	5		excluded from analysis -> deviant amplification curve						
	1	10^7 spores 2 hours H_2O + tissue fire salamander	28.68	27.98	30.76	25.51	21.91	26.5	23.48
	2		28.42	27.61	30.63	24.71	21.59	25.84	23.5
	3		28.12	27.07	29.47	24.21	20.67	25.22	22.41
	4		28.85	28.21	31.21	25.58	21.83	26.16	23.65
	5		28.19	27.44	29.22	24.62	21.08	26.02	23.03
experiment 3	1	spores	27.31	28.41	28.97	21.89	19.61	24.09	21.00
	2		26.64	28.86	28.6	21.53	19.42	23.4	20.48
	3		27.46	30.00	30.29	22.94	21.08	24.05	21.47
	4		27.55	30.02	30.3	22.88	21.01	24.32	21.63
	5		24.59	26.34	26.92	20.41	17.69	22.92	19.37
	6		24.93	25.8	27.28	20.87	18.15	22.94	19.33
	1	*Bsal*-infected skin (chin) of fire salamander 1	28.97	29.29	33.63	27.02	24.96	28.54	25.11
	2	*Bsal*-infected skin (belly) of fire salamander 1	29.16	31.84	ND	27.2	27.89	29.75	28.07
	3	*Bsal*-infected skin (tail) of fire salamander 1	30.75	31.82	34.67	28.89	29.19	30.32	28.76
	4	*Bsal*-infected skin (chin) of fire salamander 2	31.06	ND	ND	29.17	28.78	30.05	28.12
	5	*Bsal*-infected skin (belly) of fire salamander 2	30.33	30.94	ND	27.97	27.33	29.21	27.21
	6	*Bsal*-infected skin (tail) of fire salamander 2	29.44	29.65	33.52	26.88	25.21	28.57	25.12
	7	*Bsal*-infected skin (chin) of fire salamander 3	30.5	31.69	ND	27.6	27.56	30.94	29.26
	8	*Bsal*-infected skin (belly) of fire salamander 3	ND	ND	ND	ND	ND	ND	ND
	9	*Bsal*-infected skin (tail) of fire salamander 3	ND	ND	ND	ND	ND	ND	32.48

ND = no signal detected

Supplementary Table S2: Ranking of genes' CT variation across different growth stages of *Bd* and *Bsal*.

Bd	All growth stages: D0+D3+D5			Spores D0			Sporangia D3			Mature sporangia D5		
Rank	Gene	Mean	Stdev	Gene	Mean	Stdev	Gene	Mean	Stdev	Gene	Mean	Stdev
1	*TEF1F*	25.70	0.94	*TUB*	27.49	0.37	*TUB*	26.32	0.19	*TUB*	25.19	0.57
2	*TUB*	26.33	1.04	*APRT*	28.27	0.40	*APRT*	26.77	0.33	*APRT*	25.56	0.67
3	*APRT*	26.87	1.23	*R6046*	27.99	0.44	*Ctsyn1*	26.52	0.40	*TEF1F*	25.00	0.84
4	*R6046*	26.26	1.43	*α-centractin*	28.70	0.58	*α-centractin*	26.6	0.49	*R6046*	25.40	0.96
5	*Ctsyn1*	27.56	1.50	*Ctsyn1*	29.08	0.63	*TEF1F*	25.95	0.59	*GAPDH*	26.14	1.31
6	*α-centractin*	27.35	1.58	*GAPDH*	28.74	0.71	*R6046*	25.38	0.65	*Ctsyn1*	27.08	1.67
7	*GAPDH*	26.96	1.60	*TEF1F*	26.19	0.97	*GAPDH*	26.01	0.89	*α-centractin*	26.75	2.15
Bsal	All growth stages: D0+D3+D5			Spores D0			Sporangia D3			Mature sporangia D5		
Rank	Gene	Mean	Stdev	Gene	Mean	Stdev	Gene	Mean	Stdev	Gene	Mean	Stdev
1	*TUB*	23.47	0.72	*TUB*	23.41	0.62	*APRT*	28.40	0.40	*α-centractin*	27.69	0.61
2	*α-centractin*	27.09	0.99	*GAPDH*	20.58	0.92	*Ctsyn1*	27.43	0.47	*TUB*	23.20	0.72
3	*APRT*	28.86	1.02	*α-centractin*	26.15	1.05	*α-centractin*	27.42	0.50	*APRT*	29.67	0.79
4	*Ctsyn1*	28.07	1.30	*R6046*	21.79	1.05	*TEF1F*	26.47	0.69	*Ctsyn1*	28.89	1.05
5	*GAPDH*	23.37	2.40	*APRT*	28.51	1.26	*TUB*	23.81	0.81	*R6046*	26.23	1.13
6	*R6046*	25.12	2.79	*TEF1F*	19.57	1.42	*GAPDH*	25.49	1.40	*GAPDH*	24.05	1.22
7	*TEF1F*	23.28	3.12	*Ctsyn1*	27.88	1.76	*R6046*	27.35	1.85	*TEF1F*	23.80	1.26

Supplementary Table S3: Comprehensive ranking of *Bd* reference gene stability using different statistical algorithms. Delta CT, BestKeeper, NormFinder, GeNorm and RefFinder were used to determine reference gene stability in *Bd* spores day 0 (D0) (n = 6), sporangia day 3 (D3) (n = 6), mature sporangia day 5 (D5) (n = 6) and a combination of all life stages (D0+D3+D5) (n = 18). Candidate genes were ranked from most stable (1) to least stable (7).

		ΔCT	Bestkeeper analysis			NormFinder	GeNorm	RefFinder
D0+D3+D5	rank	SD	SD	CV	r	stability value	M value	Geomean
	1	*R6046* (0.78)	*TEF1F* (0.72)	TEF1F *(2.79)*	*GAPDH* (0.97)	*R6046* (0.27)	*APRT* (0.58)	*R6046* (1.86)
	2	*GAPDH* (0.84)	*TUB* (0.82)	*TUB* (3.10)	*R6046* (0.96)	*α-centractin* (0.30)	*TUB* (0.58)	*APRT* (2.63)
	3	*Ctsyn1* (0.86)	*APRT* (1.00)	*APRT* (3.71)	*Ctsyn1* (0.94)	*GAPDH* (0.33)	*R6046* (0.65)	*TUB* (2.66)
	4	*APRT* (0.87)	*R6046* (1.19)	*R6046 (4.52)*	*α-centractin* (0.90)	*Ctsyn1* (0.35)	*GAPDH* (0.73)	*GAPDH* (3.13)
	5	*TUB* (0.88)	*α-centractin* (1.27)	*α-centractin* (4.65)	*APRT* (0.89)	*APRT* (0.35)	*Ctsyn1* (0.78)	*Ctsyn1* (4.21)
	6	*α-centractin* (0.99)	*GAPDH* (1.29)	*GAPDH* (4.77)	*TUB* (0.88)	*TUB* (0.39)	*α-centractin* (0.83)	*TEF1F* (4.30)
Bd	7	*TEF1F* (1.08)	*Ctsyn1 (1.34)*	*Ctsyn1* (4.86)	*TEF1F* (0.77)	*TEF1F* (0.51)	*TEF1F* (0.90)	*α-centractin* (5.73)

		ΔCT	Bestkeeper analysis			NormFinder	GeNorm	RefFinder
D0	rank	SD	SD	CV	r	stability value	M value	Geomean
	1	*Ctsyn1* (0.36)	*TUB* (0.28)	*TUB* (1.01)	*Ctsyn1* (0.98)	*Ctsyn1* (0.06)	*Ctsyn1* (0.16)	*Ctsyn1* (1.50)
	2	*α-centractin* (0.38)	*APRT* (0.33)	*APRT* (1.17)	*α-centractin* (0.93)	*α-centractin* (0.14)	*α-centractin* (0.18)	*α-centractin* (2.00)
	3	*R6046* (0.41)	*R6046* (0.35)	*R6046* (1.26)	*GAPDH* (0.93)	*R6046* (0.17)	*GAPDH* (0.19)	*APRT* (3.36)
	4	*APRT* (0.41)	*α-centractin* (0.46)	*α-centractin* (1.61)	R6046 (0.89)	*APRT* (0.18)	*APRT* (0.29)	*R6046* (3.41)
	5	*GAPDH* (0.44)	*Ctsyn1* (0.53)	*Ctsyn1* (1.83)	*TEF1F* (0.89)	*GAPDH* (0.21)	*R6046* (0.32)	*TUB* (3.83)
	6	*TUB* (0.53)	*GAPDH* (0.56)	*GAPDH* (1.96)	*APRT* (0.88)	*TUB* (0.32)	*TUB* (0.37)	*GAPDH* (4.61)
Bd	7	*TEF1F* (0.70)	*TEF1F* (0.80)	*TEF1F* (3.04)	*TUB* (0.64)	*TEF1F* (0.45)	*TEF1F* (0.46)	*TEF1F* (7.00)

		ΔCT	Bestkeeper analysis			NormFinder	GeNorm	RefFinder
D3	rank	SD	SD	CV	r	stability value	M value	Geomean
	1	*Ctsyn1* (0.48)	*TUB* (0.14)	*TUB* (0.54)	*R6046* (0.95)	*Ctsyn1* (0.15)	*α-centractin* (0.36)	*Ctsyn1* (1.32)
	2	*α-centractin* (0.52)	*APRT* (0.24)	*APRT* (0.91)	*TEF1F* (0.90)	*α-centractin* (0.21)	*Ctsyn1* (0.37)	*α-centractin* (2.11)
	3	*TEF1F* (0.52)	*Ctsyn1* (0.27)	*Ctsyn1* (1.03)	*GAPDH* (0.89)	*TEF1F* (0.21)	*R6046* (0.42)	*TEF1F* (3.46)
	4	*R6046* (0.52)	*TEF1F* (0.40)	*α-centractin* (1.52)	*Ctsyn1* (0.79)	*R6046* (0.23)	*TEF1F* (0.44)	*TUB* (3.66)
	5	*APRT* (0.62)	*α-centractin* (0.40)	*TEF1F* (1.53)	*α-centractin* (0.78)	*TUB* (0.36)	*APRT* (0.49)	*R6046* (4.12)
	6	*TUB* (0.64)	*R6046* (0.43)	*R6046* (1.69)	*APRT* (0.11)	*APRT* (0.36)	*TUB* (0.52)	*APRT* (4.16)
Bd	7	*GAPDH* (0.71)	*GAPDH* (0.57)	*GAPDH (2.20)*	*TUB* (0.30)	*GAPDH* (0.46)	*GAPDH* (0.57)	*GAPDH* (7.00)

D5	rank	ΔCT	Bestkeeper analysis			NormFinder	GeNorm	RefFinder
		SD	SD	CV	r	stability value	M value	Geomean
	1	*R6046* (0.70)	*TUB* (0.41)	*TUB* (1.64)	*GAPDH* (0.98)	*GAPDH* (0.13)	*APRT* (0.30)	*TUB* (2.24)
	2	*TEF1F* (0.72)	*APRT* (0.51)	*APRT* (2.01)	*Ctsyn1* (0.92)	*R6046* (0.16)	*TUB* (0.33)	*R6046* (2.38)
	3	*GAPDH* (0.74)	*TEF1F* (0.60)	*TEF1F* (2.40)	*R6046* (0.96)	*TEF1F* (0.29)	*TEF1F* (0.34)	*APRT* (2.38)
	4	*APRT* (0.81)	*R6046 (0.65)*	*R6046 (2.57)*	*TUB* (0.93)	*APRT* (0.45)	*R6046* (0.39)	*TEF1F* (2.71)
	5	*TUB* (0.82)	*GAPDH* (0.85)	*GAPDH* (3.26)	*TEF1F* (0.93)	*TUB* (0.45)	*GAPDH* (0.49)	*GAPDH* (2.94)
	6	*Ctsyn1* (0.98)	*Ctsyn1* (1.30)	*Ctsyn1* (4.78)	*α-centractin* (0.92)	*Ctsyn1* (0.49)	*Ctsyn1* (0.66)	*Ctsyn1* (6.00)
Bd	7	*α-centractin* (1.46)	*α-centractin* (1.49)	*α-centractin* (5.56)	*APRT* (0.88)	*α-centractin* (0.97)	*α-centractin* (0.89)	*α-centractin* (7.00)

The differences in rankings are open to interpretation by the scientific community and their selection of reference genes according to their preferred statistical algorithm.

Supplementary Table S4: Comprehensive ranking of *Bsal* reference gene stability using different statistical algorithms. Delta CT, BestKeeper, NormFinder, GeNorm and RefFinder were used to determine reference gene stability in *Bsal* spores day 0 (D0) (n = 6), sporangia day 3 (D3) (n = 6), mature sporangia day 5 (D5) (n = 6) and a combination of all life stages (D0+D3+D5) (n = 18). Candidate genes were ranked from most stable (1) to least stable (7).

D0+D3+D5	rank	ΔCT	Bestkeeper analysis			NormFinder	GeNorm	RefFinder
		SD	SD	CV	r	stability value	M value	Geomean
	1	*α-centractin* (1.47)	*TUB* (0.59)	*TUB* (2.50)	*GAPDH* (0.96)	*α-centractin* (0.45)	*APRT* (0.55)	*α-centractin* (1.57)
	2	*APRT* (1.69)	*α-centractin* (0.70)	*α-centractin* (2.60)	*R6046* (0.96)	*GAPDH* (0.75)	*Ctsyn1* (0.61)	*APRT* (2.21)
	3	*GAPDH* (1.72)	*APRT* (0.79)	*APRT* (2.72)	*TEF1F* (0.94)	*APRT* (0.85)	*α-centractin* (0.68)	*TUB* (2.63)
	4	*TUB* (1.78)	*Ctsyn1* (0.93)	*Ctsyn1* (3.32)	*α-centractin* (0.92)	*TUB* (0.85)	*TUB* (0.87)	*Ctsyn1* (3.16)
	5	*Ctsyn1* (1.86)	*GAPDH* (2.01)	*GAPDH* (8.60)	*TUB* (0.58)	*R6046* (1.03)	*GAPDH* (1.39)	*GAPDH* (3.50)
	6	*R6046* (1.97)	*R6046* (2.34)	*R6046* (9.30)	*APRT* (0.53)	*Ctsyn1* (1.04)	*R6046* (1.64)	*R6046* (6.00)
Bsal	7	*TEF1F*(2.28)	*TEF1F* (2.63)	*TEF1F* (11.30)	*Ctsyn1* (0.43)	*TEF1F* (1.16)	*TEF1F* (1.82)	*TEF1F* (7.00)

D0	rank	ΔCT	Bestkeeper analysis			NormFinder	GeNorm	RefFinder
		SD	SD	CV	r	stability value	M value	Geomean
	1	*α-centractin* (0.43)	*TUB* (0.48)	*TUB* (2.05)	*GAPDH* (0.99)	*α-centractin* (0.09)	*GAPDH* (0.27)	*α-centractin* (1.86)
	2	*R6046* (0.46)	*GAPDH* (0.74)	*α-centractin* (3.22)	*α-centractin* (0.99)	*R6046* (0.13)	*α-centractin* (0.28)	*R6046* (1.86)
	3	*GAPDH* (0.47)	*R6046* (0.80)	*APRT* (3.56)	*TEF1F* (0.99)	*APRT* (0.15)	*R6046* (0.28)	*GAPDH* (2.21)
	4	*APRT* (0.48)	*α-centractin* (0.85)	*GAPDH* (3.57)	*Ctsyn1* (0.99)	*GAPDH* (0.18)	*APRT* (0.33)	*TUB* (3.83)
	5	*TEF1F* (0.56)	*APRT* (1.02)	*R6046* (3.68)	*R6046* (0.98)	*TEF1F* (0.27)	*TEF1F* (0.39)	*APRT* (3.94)
	6	*TUB* (0.71)	*TEF1F* (1.14)	*Ctsyn1* (4.82)	*APRT* (0.98)	*TUB* (0.46)	*TUB* (0.46)	*TEF1F* (5.23)
Bsal	7	*Ctsyn1* (0.80)	*Ctsyn1* (1.35)	*TEF1F* (5.83)	*TUB* (0.95)	*Ctsyn1* (0.52)	*Ctsyn1* (0.56)	*Ctsyn1* (7.00)

D3	rank	ΔCT	Bestkeeper analysis			NormFinder	GeNorm	RefFinder
		SD	SD	CV	r	stability value	M value	Geomean
	1	*TEF1F* (0.76)	*APRT* (0.28)	*APRT* (0.97)	*GAPDH* (0.97)	*TEF1F* (0.15)	*Ctsyn1* (0.22)	*TEF1F* (2.00)
	2	*α-centractin* (0.79)	*Ctsyn1* (0.37)	*Ctsyn1* (1.34)	*R6046* (0.93)	*TUB* (0.21)	*APRT* (0.23)	*APRT* (2.11)
	3	*TUB* (0.83)	*α-centractin* (0.42)	*α-centractin* (1.52)	*TEF1F* (0.92)	*α-centractin* (0.36)	*α-centractin* (0.24)	*Ctsyn1* (2.51)
	4	*Ctsyn1* (0.88)	*TEF1F* (0.51)	*TEF1F* (1.93)	*TUB* (0.89)	*APRT* (0.51)	*TEF1F* (0.43)	*α-centractin* (2.71)
	5	*APRT* (0.88)	*TUB* (0.66)	*TUB* (2.76)	*α-centractin* (0.69)	*Ctsyn1* (0.52)	*TUB* (0.56)	*TUB* (3.50)
	6	*GAPDH* (1.05)	*GAPDH* (0.98)	*GAPDH* (3.83)	*Ctsyn1* (0.38)	*GAPDH* (0.55)	*GAPDH* (0.75)	*GAPDH* (6.00)
Bsal	7	*R6046* (1.45)	*R6046* (1.38)	*R6046* (5.06)	*APRT* (0.35)	*R6046* (0.96)	*R6046* (0.95)	*R6046* (7.00)

D5	rank	ΔCT SD	Bestkeeper analysis SD	Bestkeeper analysis CV	Bestkeeper analysis r	NormFinder stability value	GeNorm M value	RefFinder Geomean
	1	*TUB* (0.54)	*α-centractin* (0.53)	*α-centractin* (1.91)	*TUB* (0.99)	*TUB* (0.09)	*TUB* (0.27)	*TUB* (1.19)
	2	*APRT* (0.63)	*TUB* (0.55)	*APRT* (2.08)	*TEF1F* (0.94)	*α-centractin* (0.26)	*α-centractin* (0.30)	*α-centractin* (1.57)
	3	*α-centractin* (0.64)	*APRT* (0.62)	*TUB* (2.38)	*α-centractin* (0.93)	*APRT* (0.28)	*APRT* (0.32)	*APRT* (2.71)
	4	*R6046* (0.75)	*Ctsyn1* (0.85)	*Ctsyn1* (2.94)	*GAPDH* (0.92)	*R6046* (0.39)	*GAPDH* (0.54)	*R6046* (4.47)
	5	*GAPDH* (0.77)	*R6046* (0.90)	*R6046* (3.43)	*R6046* (0.91)	*GAPDH* (0.43)	*R6046* (0.61)	*GAPDH* (5.14)
	6	*TEF1F* (0.78)	*TEF1F* (0.92)	*TEF1F* (3.88)	*APRT* (0.88)	*TEF1F* (0.43)	*TEF1F* (0.64)	*TEF1F* (6.00)
Bsal	7	*Ctsyn1* (0.87)	*GAPDH* (0.99)	*GAPDH* (4.13)	*Ctsyn1* (0.74)	*Ctsyn1* (0.55)	*Ctsyn1* (0.71)	*Ctsyn1* (6.09)

The differences in rankings are open to interpretation by the scientific community and their selection of reference genes according to their preferred statistical algorithm.

Supplementary Table S5: Descriptive statistics of 7 *Bd* and *Bsal* candidate reference genes' expression based on the BestKeeper approach.

			TEF1F	***α-centractin***	***Ctsyn1***	***GAPDH***	***R6046***	***TUB***	***APRT***
Bd	All growth stages: D0+D3+D5	geo Mean [CP]	25.68	27.31	27.52	26.92	26.22	26.31	26.83
		min [CP]	23.41	24.58	25.02	23.87	23.66	24.19	24.42
		max [CP]	27.61	30.7	29.96	29.91	28.57	28.11	28.83
		std dev [+/- CP]	0.72	1.27	1.34	1.29	1.19	0.82	1.00
		CV [% CP]	2.79	4.65	4.86	4.77	4.52	3.10	3.71
		coeff. of corr. [r]	0.753	0.90	0.94	0.97	0.962	0.88	0.89
	Fresh spores D0	geo Mean [CP]	26.16	28.69	29.07	28.73	27.99	27.49	28.27
		min [CP]	25.27	27.98	28.47	28.06	27.41	27.11	27.76
		max [CP]	27.61	29.41	29.96	29.91	28.57	28.11	28.83
		std dev [+/- CP]	0.80	0.46	0.53	0.56	0.352	0.28	0.33
		CV [% CP]	3.04	1.61	1.83	1.96	1.26	1.01	1.17
		coeff. of corr. [r]	0.89	0.93	0.981	0.934	0.888	0.64	0.88
	Sporangia D3	geo Mean [CP]	25.95	26.59	26.51	26.00	25.37	26.32	26.77
		min [CP]	24.76	26.15	26.14	24.29	24.24	26.08	26.47
		max [CP]	26.3	27.29	27.24	26.87	26.2	26.58	27.37
		std dev [+/- CP]	0.40	0.40	0.27	0.57	0.43	0.14	0.24
		CV [% CP]	1.53	1.52	1.03	2.20	1.69	0.54	0.91
		coeff. of corr. [r]	0.90	0.78	0.79	0.89	0.95	-0.30	0.11
	Mature sporangia D5	geo Mean [CP]	24.94	26.68	27.03	26.11	25.39	25.18	25.55
		min [CP]	23.41	24.58	25.02	23.87	23.66	24.19	24.42
		max [CP]	25.84	30.70	29.56	27.71	26.54	25.79	26.28
		std dev [+/- CP]	0.60	1.49	1.30	0.85	0.65	0.41	0.51
		CV [% CP]	2.40	5.56	4.78	3.26	2.57	1.64	2.01
		coeff. of corr. [r]	0.93	0.92	0.97	0.98	0.96	0.93	0.88
Bsal	All growth stages: D0+D3+D5	geo Mean [CP]	23.07	27.07	28.04	23.26	24.97	23.46	28.84
		min [CP]	17.71	24.82	25.74	19.49	20.44	22.41	26.97
		max [CP]	27.18	28.43	30.73	26.82	29.02	24.63	30.99
		std dev [+/- CP]	2.63	0.70	0.93	2.01	2.34	0.59	0.79

		CV [% CP]	11.30	2.60	3.33	8.60	9.30	2.50	2.72
		coeff. of corr. [r]	0.94	0.915	0.433	0.961	0.956	0.579	0.527
	Fresh spores D0	geo Mean [CP]	19.52	26.13	27.84	20.56	21.77	23.40	28.49
		min [CP]	17.71	24.82	25.74	19.49	20.44	22.62	26.97
		max [CP]	21.04	27.21	29.88	21.73	23.26	24.28	29.85
		std dev [+/- CP]	1.14	0.84	1.35	0.735	0.80	0.48	1.02
		CV [% CP]	5.83	3.22	4.82	3.57	3.68	2.05	3.56
		coeff. of corr. [r]	0.99	0.99	0.99	0.99	0.98	0.95	0.98
	Sporangia D3	geo Mean [CP]	26.46	27.42	27.43	25.46	27.29	23.80	28.39
		min [CP]	25.21	26.87	26.73	22.94	24.08	22.52	27.86
		max [CP]	27.18	28.09	28.00	26.82	29.02	24.63	29.06
		std dev [+/- CP]	0.51	0.42	0.37	0.98	1.38	0.66	0.28
		CV [% CP]	1.93	1.52	1.34	3.83	5.06	2.76	0.97
		coeff. of corr. [r]	0.92	0.69	0.38	0.97	0.93	0.89	0.35
	Mature sporangia D5	geo Mean [CP]	23.77	27.68	28.87	24.02	26.21	23.19	29.66
		min [CP]	21.87	26.94	28.08	22.67	25.25	22.41	28.78
		max [CP]	25.53	28.43	30.73	25.59	28.07	24.4	30.99
		std dev [+/- CP]	0.92	0.53	0.85	0.99	0.90	0.55	0.62
		CV [% CP]	3.88	1.91	2.94	4.13	3.43	2.38	2.08
		coeff. of corr. [r]	0.94	0.93	0.74	0.92	0.91	0.99	0.88

Supplementary Table S6: Summary of the suggested reference genes. Based on the obtained results and the different statistical algorithms used in the manuscript (Delta CT, BestKeeper, NormFinder, GeNorm and RefFinder), a summary is made of possible reference gene combinations. Both for *Bd* and *Bsal* an overview is given of what reference genes could be used when assessing target gene expression in spores day 0 (D0), sporangia day 3 (D3), mature sporangia day 5 (D5) and in *Bd* or *Bsal*-infected skin tissue.

	number	*Bd*	*Bsal*
Spores D0	1	*Ctsyn1*	*GAPDH*
	2	*α-centractin*	*α-centractin*
Sporangia D3	1	*α-centractin*	*Ctsyn1*
	2	*Ctsyn1*	*APRT*
	3	*R6046*	
Mature sporangia D5	1	*APRT*	*TUB*
	2	*TUB*	*α-centractin*
Infected tissue (*in vivo*)	1	*APRT*	*GAPDH*
	2	*TUB*	*TUB*
	3	*R6046*	*R6046*
	4	*GAPDH*	*α-centractin*

Supplementary Table S7: List of genes and sequences of the primers used for quantitative PCR analysis.

	Gene	Forward primer	Reverse primer	Amplicon size (bp)	E (%)	R^2	Slope	Melt T	Ref
	α-centractin	GCAGCATGGAGTTGTCACTG	AGCTTGGTCACGATTGGAAC	145	91,9	0,997	-3,533	81,5	[5]
	Ctsyn1	TCCTCAGCAGCTCCTATTCG	CTCGACGTCCTTTTTCAGGA	262	93,3	0,998	-3,493	82,5	this manuscript
	APRT	GGTTGCCACTTGGAGTCTGT	ATGGCTGGATGGAAACTCTG	97	96,9	0,998	-3,399	81,0	this manuscript
	R6046	GTCGTACTGGCAACCTCACC	ACATTGGGAGCAATCTCGAC	79	96	0,997	-3,422	81,0	[5]
	TEF1F	CCTTCCCGTCCTACTGACAA	GAACAGTTCCGATTCCTCCA	76	93,7	0,999	-3,484	79,5	[5]
	TUB	CTCTCGGTGGTGGTACTGGT	AGGGTATTCCTCGCGAATCT	68	100,3	0,994	-3,315	80,0	this manuscript
Bd	*GAPDH*	AAGCCTGCCAAGTACGAAGA	AAAGATGGAGCTGCGAGTGT	135	94,3	0,995	-3,466	84,0	[5]
	α-centractin	CCGGCTACCATTTTCATACG	CGATCGATGGGTAGCACTCT	116	97,9	0,995	-3,375	80,0	[5]
	Ctsyn1	CGCCAGAAGCATCTCTTTTC	AACTCGTCCCAGTTGTCAGC	149	99,6	0,986	-3,332	83,5	this manuscript
	APRT	GGAAACAGACCCAGCTACCA	GTTCCGACCATATCCACCAC	99	87,2	0,997	-3,672	84,0	this manuscript
	R6046	GTTGCCAAGTCTGCTGTGAA	ATCAAGCGAGGGTGCAGAC	87	90,2	0,997	-3,582	81,5	[5]
	TEF1F	TCCCACTGACAAACCTCTCC	CGACAGGTACTGTTCCAATACCAC	74	95,3	0,994	-3,439	78,5	[5]
	TUB	AAGAGCCAACGACACCAATC	GAAGGACTGGGTGCTGAAGA	99	99	0,995	-3,347	79,5	this manuscript
Bsal	*GAPDH*	GCCAGCAAAATACGAGGAGA	CCATTCATGGGTCCATTAGC	63	94	0,999	-3,475	77,5	[5]
